# Transcriptional and cellular signatures of cortical morphometric remodelling in chronic pain

**DOI:** 10.1097/j.pain.0000000000002480

**Published:** 2021-09-23

**Authors:** Daniel Martins, Ottavia Dipasquale, Mattia Veronese, Federico Turkheimer, Marco L. Loggia, Stephen McMahon, Matthew A. Howard, Steven C.R. Williams

**Affiliations:** a Department of Neuroimaging, Institute of Psychiatry, Psychology and Neuroscience, King's College London, London, United Kingdom; b Department of Radiology, Athinoula A. Martinos Center for Biomedical Imaging, Harvard Medical School, Massachusetts General Hospital Boston, MA, United States; c Wolfson CARD, Institute of Psychiatry, Psychology and Neuroscience, King's College London, London, United Kingdom

**Keywords:** Chronic pain, Morphometric similarity, Neuroinflammation, Allen Brain Atlas, Transcriptomics

## Abstract

Chronic pain is a highly debilitating and difficult to treat condition, which affects the structure of the brain. Although the development of chronic pain is moderately heritable, how disease-related alterations at the microscopic genetic architecture drive macroscopic brain abnormalities is currently largely unknown. Here, we examined alterations in morphometric similarity (MS) and applied an integrative imaging transcriptomics approach to identify transcriptional and cellular correlates of these MS changes, in 3 independent small cohorts of patients with distinct chronic pain syndromes (knee osteoarthritis, low back pain, and fibromyalgia) and age-matched and sex-matched pain-free controls. We uncover a novel pattern of cortical MS remodelling involving mostly small-to-medium MS increases in the insula and limbic cortex (none of these changes survived stringent false discovery rate correction for the number of regions tested). This pattern of changes is different from that observed in patients with major depression and cuts across the boundaries of specific pain syndromes. By leveraging transcriptomic data from Allen Human Brain Atlas, we show that cortical MS remodelling in chronic pain spatially correlates with the brain-wide expression of genes related to pain and broadly involved in the glial immune response and neuronal plasticity. Our findings bridge levels to connect genes, cell classes, and biological pathways to in vivo imaging correlates of chronic pain. Although correlational, our data suggest that cortical remodelling in chronic pain might be shaped by multiple elements of the cellular architecture of the brain and identifies several pathways that could be prioritized in future genetic association or drug development studies.

## Abstract 2

Supplemental Digital Content is Available in the Text.

Imaging transcriptomics bridge levels to connect genes, cell classes, and biological pathways to in vivo imaging correlates of cortical morphometric remodelling chronic pain.

## 1. Introduction

Chronic pain is a prevalent and highly debilitating condition^11,23,61^ with a moderately strong genetic basis (heritability, h^2^ ≈ 16%-50%).^34,67^ Treatment response to current standard treatments is complex and overall poor.^24^ Identifying new potential targets for drug development has become over the years a priority for researchers and clinicians. However, therapeutic advances have been marred by our still poor understanding of the mechanisms underlying chronic pain.^85^

Chronic pain has been increasingly recognized as a disorder of the brain.^12,22,39,66,78,89^ Using different neuroimaging techniques, a rich body of evidence has shown that most chronic pain syndromes is associated with a number of spatially patterned structural and functional alterations in different cortical and subcortical regions and brainstem.^40,58,75,89^ The aberrant functional^19,26,41,48^ and structural^10,21,45,92^ configuration of the brain network detected across patients with different chronic pain syndromes has given rise to the idea that this distributed pattern of brain changes might reflect disruption of large-scale brain networks comprising anatomically connected brain areas.^25^ However, exploring this hypothesis further has been constrained by challenges in measuring anatomical connectivity in humans.^5^ For instance, the use of diffusion-weighted imaging to estimate the connectivity of long-distance projections, such as those between hemispheres, remains challenging.^28^ On the other hand, structural covariance analysis is not applicable at the single-subject level, and because it relies on group-based covariance, it requires large sample sizes to be reliably estimated.^5^

Morphometric similarity (MS) mapping has recently emerged as a new approach to construct whole-brain anatomical networks for individual subjects, overcoming some of the methodological limitations highlighted above.^62,76,77^ It quantifies the similarity between cortical regions for multiple magnetic resonance imaging (MRI) parameters measured in each area.^77^ This metric has close associations with the cytoarchitectonic properties of the cortex and axonal connectivity between regions.^77^ Morphometric similarity mapping is a reliable method that can capture interindividual differences in cognition^77^ and clinical abnormalities in brain disorders.^52,62,76^ However, its use for uncovering morphometric differences in the brain of patients with chronic pain syndromes remains untried.

Spatially diffuse correlates of chronic pain across cortical anatomy could arise from a host of biological changes in patients, such as altered neurotransmission and maladaptive synaptic plasticity^44,60,70,72^ and neuroinflammation,^37,38,90,94^ among others. However, most neuroimaging modalities are not sensitive to underlying molecular or transcriptional properties of brain tissue. Therefore, understanding how disease-related alterations at the microscopic transcriptional architecture might explain regional vulnerability to macroscopic brain abnormalities in chronic pain syndromes remains rather challenging.

**Figure 1.**
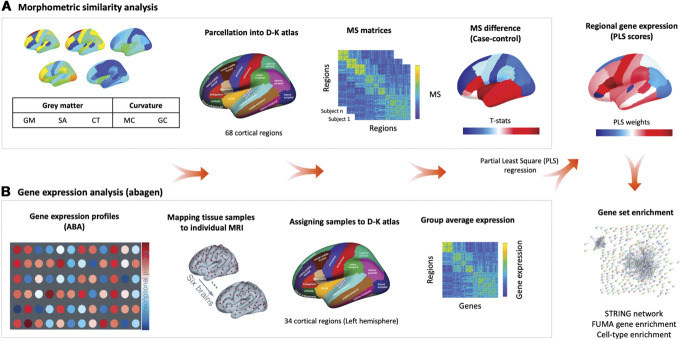
Overview of the analysis pipeline. (A) Morphometric similarity (MS) analysis. We constructed individual cortical MS matrices using 5 structural magnetic resonance imaging features (eg, gray matter volume [GM], surface area [SA], cortical thickness [CT], mean curvature [MC], and Gaussian curvature [GC]) extracted from 68 cortical regions of the Desikan–Killiany (DK) Atlas. For each individual, we produced a 68 × 68 MS matrix by correlating the normalized (z-scores) values of the 5 structural features between each pair of regions in the atlas. For each region, we averaged across all the edges involving that area to obtain a singular representation of the mean MS score for that region. We then computed case–control differences for each region, while accounting for age, gender, and total intracranial volume. (B) Gene expression analysis. We used abagen to obtain gene expression profiles from the Allen Human Brain Atlas (AHBA) in 68 regions (left hemisphere only) across the 6 postmortem brains sampled in this atlas. We excluded all genes with normalized expression values below the background (15,633 genes met this criterion). When more than one probe was available for a certain gene, we selected the probe with higher consistency in expression across the 6 donors. We used partial least squares regression (PLS) to rank all genes according to their association with the case–control changes in MS. Finally, we performed a set of enrichment analyses on the top genes positively or negatively associated with case–control differences in MS.

Here, we sought to bridge these gaps by examining alterations in MS in 3 independent case–control studies of patients with chronic pain syndromes: knee osteoarthritis (OA), chronic low back pain (CLBP), and fibromyalgia (FM). Moreover, given the tight relationship between regional MS and gene expression,^77^ we leveraged data from the Allen Human Brain Atlas (AHBA) (Fig. 1) to explore among potential molecular and cellular pathways that might explain regional vulnerability to MS changes during chronic pain.

## 2. Methods

### 2.1. Samples

We used structural MRI data from 3 prior case–control studies on knee OA,^86^ CLBP,^56^ and FM.^69^ The full details of each sample (including inclusion and exclusion criteria) have been described in the original publications. The OA data set included 56 OA chronic pain patients (54% women, 58 ± 6.96 years) and 20 age-matched healthy control subjects (50% women, 58 ± 6.65 years). The CLBP data set included 29 CLBP patients (59% women, 30.79 ± 11.59 years) and 33 healthy controls (14 females, 31.18 ± 9.65 years). The FM data set included 20 women with FM (46.4 ± 12.4 years) and 20 age-matched healthy control women (42.1 ± 12.5 years). The original studies were approved by the competent ethics assessment board of each respective host institution. All participants provided informed consent before enrolling the respective studies.

### 2.2. MRI data acquisition

We used high-resolution T1-weighted anatomical images acquired in 3T scanners. We provide a summary of the parameters used for T1-weighted anatomical images acquisition in each data set (as described in the original publications).

#### 2.2.1. Osteoarthritis data set

Images were acquired in a Siemens 3.0 Trio whole-body scanner using the standard radio-frequency head coil with the following parameters: TR/TE = 2500/3.36 milliseconds, flip angle = 9°, in-plane matrix resolution = 256 × 256, FOV = 256 mm^2^, slice thickness = 1 mm, and number of slices = 160.^86^

#### 2.2.2. Chronic low back pain data set

Images were acquired in a Siemens 3.0 T Trio B whole-body scanner equipped with a 32-channel head coil with the following parameters: TR/TE = 1900/2.52 milliseconds, flip angle = 9°, matrix 256 × 256, slice thickness = 1 mm, and number of slices = 176.^56^

#### 2.2.3. Fibromyalgia data set

Images were acquired in a 3.0 Tesla GE Discovery MR750 scanner (HD, General Electric Healthcare, Waukesha, WI) and a commercial 32-channel head coil array, using the FSPGR BRAVO pulse sequence: TR/TE = 7.7/3.2 milliseconds, flip angle = 12°, matrix = 256 × 256, FOV = 256 mm^2^, slice thickness = 1 mm, and number of slices = 168.^69^

### 2.3. Morphometric similarity mapping

The T1-weighted MRI data from all participants were preprocessed using the recon-all command from FreeSurfer (version 6.0).^15^ The surfaces were then parcellated using the 68 cortical regions of the Desikan–Killiany Atlas.^20^ For each cortical region, we estimated 5 parameters: gray matter volume, surface area, cortical thickness, Gaussian curvature, and mean curvature. Each parameter was normalized for sample mean and standard deviation to account for variation in value distributions between the features. After normalization, MS networks were generated by computing the regional pairwise Pearson correlations in morphometric feature sets, yielding a 68 × 68 MS matrix *ℳ*i for each participant, i = 1, …, N, which represents the strength of MS between each pair of cortical areas. For all individuals, regional MS (ie, nodal similarity) estimates were calculated as the average MS between a given cortical region and all others. Although MS calculation allows for the inclusion of data from different imaging modalities, here, we used only features extracted from T1-weighted MRI data. It has been previously demonstrated that there is high spatial concordance (*r* = 0.91) between the topography of regional MS derived from T1-weighted MRI data alone and regional MS from more modalities (eg, a combination of T1-weighted and diffusion-weighted imaging).^43,77^

### 2.4. Case–control analysis of morphometric similarity networks

The global MS for each participant is the average of *ℳ*_i_. The regional MS_i,j_ for the ith participant at each region j = 1,…,68 is the average of the jth row (or column) of *ℳ*_i_. Thus, regional MS is equivalent to the weighted degree or “hubness” of each regional node, connected by signed and weighted edges of pair-wise similarity to all other nodes in the whole-brain connectome represented by the MS matrix. For global and regional MS statistics alike, we fit linear models to estimate case–control differences, with age, sex, and total intracranial volume as covariates. The resulting *P* value for each region was thresholded for significance using false discovery rate (FDR) < 0.05, to control type 1 error over multiple (n = 68) tests. To test for the differential contribution of single cortical features to the observed regional MS changes in each chronic pain condition, we recomputed condition-specific MS change maps with exclusion of each individual cortical feature before MS calculation and then determined which of these single-feature exclusions most change the topography of the observed MS changes. We did so by calculating pairwise Spearman correlations between the original and all other leave-one-out maps of MS changes, in each condition separately.

### 2.5. Cortical morphometric similarity remodelling in major depressive disorder and its association with chronic pain

Chronic pain is often comorbid with major depressive disorder (MDD)^8^ and these 2 entities share brain mechanisms of neuroplasticity.^80^ We used another openly available data set (see the original article for further details^50^) including high resolution structural brain data from 19 unmedicated patients with MDD (11 females, 29.45 ± 11.26 years) and 20 age-matched healthy controls (11 females, 33.52 ± 14.07 years). We characterized changes in regional MS associated with MDD and investigated whether changes in regional MS in MDD can predict those we observed in chronic pain patients. We calculated MS using the procedures described above and tested for case–control differences between MDD patients and healthy controls using linear models, where we accounted for age, sex, and intracranial volume. Finally, we calculated pairwise Spearman correlations between changes in regional MS in MDD and those observed in the different chronic pain conditions.

### 2.6. Mapping case–control differences in regional morphometric similarity to established patterns of cytoarchitectonic cortical organization

To help us to contextualize the case–control differences in regional MS we observed for the different chronic pain conditions, we mapped them in relation to well-established patterns of cytoarchitectonic organization of the cortex. To that end, we used the *von Economo* Atlas of the cortex classified by the cytoarchitectonic criteria.^95^ For each subject, we quantified MS within each parcel of these atlases and then performed case–control comparisons using linear models, with age, gender, and intracranial volume as covariates.

### 2.7. Morphometric similarity hubs susceptibility

We investigated relationships between case–control differences in regional MS and the typical pattern of regional MS distribution in healthy controls with Spearman correlations. In keeping with histological results indicating that cytoarchitectonically similar areas of the cortex are more likely to be anatomically connected and that MS in the macaque cortex was correlated with tract-tracing measurements of axonal connectivity,^77^ we followed the approach suggested by Seidlizt et al.^76^ to map each region to one of 4 patterns of changes in MS: (1) regions of low MS in healthy controls (highly differentiated from the rest of the cortex) that increase their MS during with the rest of the cortex during chronic pain (dedifferentiation), (2) regions of high MS in healthy controls (highly connected with the rest of the cortex) that increase their MS during chronic pain (hypercoupling), (3) regions of low MS in healthy controls that decrease their MS during chronic pain (hyperdifferentiation), and (4) regions of high MS in healthy controls that decrease their MS during chronic pain (decoupling). We subdivided each axis of the scatter plot in 2 sections, one above and another below 0, which resulted in 4 quadrants, each representing one of the 4 scenarios presented above. We then quantified the percentage of regions falling within each of these 4 quadrants to identify dominant patterns of change.

### 2.8. Defining a cross-condition pattern of changes in regional morphometric similarity during chronic pain

We investigated the similarity in case–control changes in regional MS across chronic pain conditions by calculating pairwise Spearman correlations of regional case–control statistics (Z-scores) from each condition.^96^ We found significant correlations for all possible pairs of conditions, which indicated that remodelling of regional MS during chronic pain shares a common pattern across conditions. We then ran principal component analysis on the 3 vectors of case–control changes in regional MS to identify this shared profile of cross-condition changes. The first component alone explained 64.45% of the shared variance, the second 25.38%, and the third 10.16%. Only the first component showed an eigenvalue > 1 (1.93). Hence, we kept only the first PC because case–control changes in regional MS across our 3 chronic pain conditions seem to be well captured by one single dominant cross-condition pattern.

### 2.9. Microarray expression data: Allen Human Brain Atlas

Regional microarray expression data were obtained from 6 postmortem brains provided by the AHBA (AHBA; http://human.brain-map.org/) (aged 24-57 years).^33^ We used the *abagen* toolbox (https://github.com/netneurolab/abagen) to process and map the transcriptomic data to 84 parcellated brain regions from the Desikan–Killiany Atlas.^20^ In brief, genetic probes were reannotated using information provided by Arnatkeviciute et al.,^7^ instead of the default probe information from the AHBA data set, hence discarding probes that cannot be reliably matched to genes. Following previously published guidelines for probe-to-gene mappings and intensity-based filtering,^7^ the reannotated probes were filtered based on their intensity relative to the background noise level; probes with intensity less than background in ≥50% of samples were discarded. A single probe with the highest differential stability, ΔS(*p*), was selected to represent each gene, where differential stability was calculated as^32^:

$$\Delta_{s}\left(p\right)=\frac{1}{\left(\begin{array}{c} N\\ 2 \end{array}\right)}\sum_{i=1}^{N-1}\sum_{j=i+1}^{N}\rho \left[B_{i}\left(p\right),B_{j}\left(p\right)\right]$$

Here, *ρ* is Spearman's rank correlation of the expression of a single probe *p* across regions in 2 donor brains, *B*_i_ and *B*_j_, and *N* is the total number of donor brains. This procedure retained 15,633 probes, each representing a unique gene.

Next, tissue samples were assigned to brain regions using their corrected MNI coordinates (https://github.com/chrisfilo/alleninf) by finding the nearest region within a radius of 2 mm. To reduce the potential for misassignment, sample-to-region matching was constrained by hemisphere and cortical or subcortical divisions. If a brain region was not assigned to any sample based on the above procedure, the sample closest to the centroid of that region was selected to ensure that all brain regions were assigned a value. Samples assigned to the same brain region were averaged separately for each donor. Gene expression values were then normalized separately for each donor across regions using a robust sigmoid function and rescaled to the unit interval. Scaled expression profiles were finally averaged across donors, resulting in a single matrix with rows corresponding to brain regions and columns corresponding to the retained 15,633 genes. As a further sanity check, we conducted leave-one-donor out sensitivity analyses to generate 6 expression maps containing gene expression data from all donors, one at a time. The principal components of these 6 expression maps were highly correlated (average Pearson correlation of 0.993), supporting the idea that our final gene expression maps where we averaged gene expression for each region across the 6 donors is unlikely to be biased by data from a specific donor. Because the AHBA only includes data for the right hemisphere for 2 subjects, in our transcriptomic-imaging association analyses, we only considered the left hemisphere cortical regions (34 regions).

### 2.10. Identifying transcriptomic correlates of cortical morphometric similarity remodelling in chronic pain

To be able to investigate associations between cross-condition changes in MS during chronic pain and brain gene expression, we used partial least square regression (PLS).^62^ Partial least square regression uses the gene expression measurements (the predictor variables) to predict the regional MS changes (the response variables). This approach allows us to rank all genes by their multivariate spatial alignment with cross-condition regional MS changes during chronic pain. The first PLS component (PLS1) is the linear combination of the weighted gene expression scores that have a brain expression map that covaries the most with the map of MS changes. As the components are calculated to explain the maximum covariance between the dependent and independent variables, the first component does not necessarily need to explain the maximum variance in the dependent variable. However, as the number of components calculated increases, they progressively tend to explain less variance in the dependent variable. Here, we tested across a range of components (between 1 and 15) and quantified the relative variance explained by each component. The statistical significance of the variance explained by each component was tested by permuting the response variables 1000 times.

In our analysis, a solution with a single component explained variance in regional MS changes above chance (*P*_boot_ = 0.003). The first PLS component (PLS1) alone explained the highest amount of variance alone (24.42%). Hence, we focused our further gene set enrichment analyses on PLS1. The error in estimating each gene's PLS1 weight was assessed by bootstrapping (resampling with replacement of the 34 brain regions), and the ratio of the weight of each gene to its bootstrap standard error was used to calculate the *Z* scores and, hence, rank the genes according to their contribution to PLS1.^95^ Genes with large positive PLS1 weights correspond to genes that have higher than average expression in regions where MS increases and lower than average expression in regions where MS decreases. Mid-rank PLS weights showed expression gradients that are weakly related to the pattern of MS changes. On the other side, genes with large negative PLS1 weights correspond to genes that have higher than average expression in regions where MS decreases the most and lower than average expression in regions where MS increases. Hence, from the ranked PLS1 list of genes, we then selected all genes with positive and negative weights Z > 3 and Z < −3, respectively (all *P*_FDR_ < 0.05, FDR corrected for the total number of genes tested). PLS1 genes with Z > 3 are for simplicity termed PLS1+ and genes with Z < −3 PLS1−. Although our choice of Z = 3 as a threshold to identify the most positively and negatively genes associated with MS changes in patients chronic pain is somehow arbitrary, we note that Z = 3 in our case corresponds to a stringent threshold of *P*_FDR_ = 0.036 (more stringent than *P*_FDR_ < 0.05). We used these 2 sets of genes for further enrichment analyses, as described below. We confirmed our enrichment analyses were not driven by the choice of this specific threshold by repeating all analyses described below considering all genes that passed a more liberal threshold of *P*_FDR_ < 0.05. The overall pattern of results did not change.

### 2.11. Protein–protein networks and gene set enrichment analysis

We then used all genes in PLS1+ and PLS1− to conduct further bioinformatics analyses investigating whether these genes map to common and relevant biological pathways. First, we used *STRING* (version 10.5)^84^ to construct protein–protein functional interactions networks. We excluded text mining as an active interaction source and used the default medium required interaction score of 0.400 to identify all possible links within our list of target genes. Second, we used the *GENE2FUNC* function from the Functional Mapping and Annotation of Genome-Wide Association Studies (*FUMA*)^93^ platform to investigate functional enrichments using rank-based Gene Ontology (GO) and Kyoto Encyclopedia of Genes and Genomes pathways enrichment analysis. We used as background all AHBA genes that passed our preprocessing criteria and hence were used in our PLS analyses (n = 15,633). We corrected for multiple gene-set enrichment testing by applying FDR correction.

### 2.12. Brain cell–type enrichment analysis

We also investigated whether our PLS1+ and PLS1− subsets of genes were particularly enriched for genes of specific brain cell types. We compiled data from 5 different single-cell studies using *postmortem* cortical samples in human postnatal subjects to avoid any bias based on the acquisition methodology or analysis or thresholding.^16,31,47,51,99^ To obtain gene sets for each cell type, categorical determinations were based on each individual study, as per the respective methods and analysis choices in the original article. All cell-type gene sets were available as part of the respective articles. We generated a single omnibus gene list for each cell type by merging the study-specific gene lists and then filtered it to retain only genes sampled in the AHBA. Two studies did not subset neurons into excitatory and inhibitory,^16,99^ and thus, those gene sets were excluded from the cell-class assignment. In addition, only one study included the annotation of the “Per” (pericyte) type, and thus, we did not consider that cell type.^47^ This approach has already been validated elsewhere.^76^

We then conducted cell-class enrichment analyses using the *GeneOverlap* package from R (version 1.26.0). We used as background all AHBA genes that passed our preprocessing criteria and hence were used in our PLS analysis (n = 15,633). *GeneOverlap* calculates the overlap between 2 sets of genes (in our case, the set of PLS1+ or PLS1−, and each of the brain cell–type omnibus gene sets derived as explained above) and uses a Fisher exact test to find whether the overlap between these 2 sets is higher than one would expect by randomly selecting a subset of genes from the background with the same number of elements. Here, enrichment is quantified as an odds ratio, where values lower or equal to 1 indicate minimal overlap between sets and hence absence of enrichment. Therefore, the null hypothesis is that the odds ratio is no larger than 1. Significant odds ratio larger than 1 indicate enrichment for genes of a specific cell type. We applied FDR correction for the number of cell types tested.

### 2.13. Pain-related and other brain disorder–related genes enrichment

We also investigated whether our PLS1+ and PLS1− subsets of genes were particularly enriched for pain-related and other brain disorder–related genes. The list of previously identified pain-related genes was defined using the following public resources: (1) the pain-related genes identified in mice gene knockout studies collected in the Pain Gene Database (http://paingeneticslab.ca/4105/06_02_pain_genetics_database.asp),^46^ (2) the pain-related genes identified in humans collected in the Human Pain Genes Database (https://humanpaingenetics.org/hpgdb),^59^ (3) the Pain Research Forum (https://www.painresearchforum.org/resources/pain-gene-resource),^17^ (4) the genes involved in human pain diseases collected in the DisGeNET (http://www.disgenet.org),^71^ and (5) the genes considered to be functioning in pain perception summarized in GO (GO:0019233). In total, we identified 2111 pain-related genes included in the above public resources. Eight hundred seven of these genes were not part of our initial list of 15,633 AHBA genes and were excluded from further analyses (please see Supplementary data S3 for the list of 1304 pain-related genes used in these analyses, available at http://links.lww.com/PAIN/B511). The lists of genes associated with other brain disorders (Alzheimer disease, Parkinson disease, Huntington disease, epilepsy, autism spectrum disorder, MDD, anxiety, bipolar disorder, and schizophrenia) were collected from the DisGeNET. We tested for gene enrichment in both PLS1+ and PLS1− subsets using *GeneOverlap*, as explained above*.*

### 2.14. Spatial permutation test (spin test)

In several analyses in the current study, we investigated the spatial correspondence between different imaging-derived measures. Although several studies have reported significance based on the assumption that the number of samples is equal to the number of regions, this is technically inaccurate, as the number of regions is both arbitrary (due to the resolution of the chosen parcellation) and non-independent (because of spatial autocorrelation among neighbouring parcels). To overcome this issue, we used spatial permutation tests (spin test) as implemented in previous studies.^5,6,91^ This approach consists in comparing the empirical correlation among 2 spatial maps to a set of null correlations, generated by randomly rotating the spherical projection of one of the 2 spatial maps before projecting it back on the brain surface. Importantly, the rotated projection preserves spatial contiguity of the empirical maps, as well as the hemispheric symmetry. Therefore, each analysis correlating values from 2 cortical maps is reported with a *P*-value derived from the spherical permutation (*P*_spin_), obtained by comparing the empirical Spearman Rho to a null distribution of 10,000 Spearman correlations, between one empirical map and the randomly rotated projections of the other map. The Matlab code to implement this test can be found in https://github.com/frantisekvasa/rotate_parcellation.

### 2.15. Data availability

Data can be accessed from open repositories in the following links (OA: https://openneuro.org/datasets/ds000208/versions/1.0.0; CLBP: http://www.openpain.org; FM: https://openneuro.org/datasets/ds001928/versions/1.1.0; MDD: https://openneuro.org/datasets/ds000171/versions/00001).

### 2.16. Code availability

The code for MS and gene expression association analyses is available at https://github.com/SarahMorgan/Morphometric_Similarity_SZ.

## 3. Results

### 3.1. Morphometric similarity is a reproducible measure in healthy controls across independent studies

The cortical maps of regional MS in Supplementary Figure S1 summarizes the anatomical distribution of areas of positive and negative MS in healthy controls from each of the 3 data sets (available at http://links.lww.com/PAIN/B508). The patterns of regional distribution of MS were highly correlated across healthy controls from the 3 data sets (Supplementary Figure S2, available at http://links.lww.com/PAIN/B508). The results are similar to those reported in other independent samples,^62,76,77^ with positive MS in frontal and temporal cortical areas (indicating high levels of similarity with the rest of the cortex) and negative MS in occipital and cingulate cortices (indicating low levels of similarity with the rest of the cortex; hence, high levels of differentiation from the rest of the cortex). This confirms the replicability of this pattern of regional MS in healthy individuals.

### 3.2. Patients with chronic pain do not differ from healthy controls in global morphometric similarity

**Figure 2.**
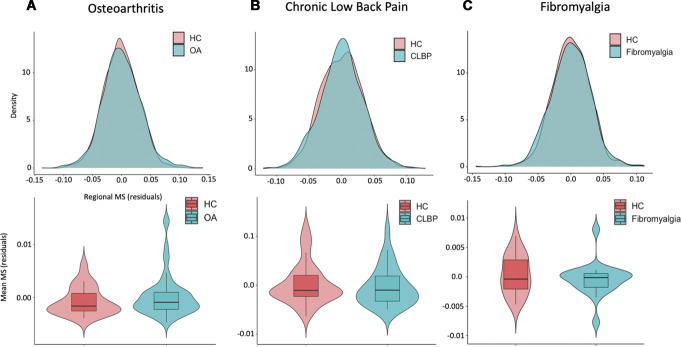
Case–control differences in global morphometric similarity. In the upper panel, we present case and control distributions of the residual regional morphometric similarity (MS) strength (ie, the average similarity of each region to all other regions) after regressing out the effects of age, gender, and intracranial volume, for each data set separately. In the lower panel, we present case–control comparisons of the global MS. To calculate global MS, we averaged the residual regional MS strength across all regions for each subject. In all 3 data sets, there were no differences between groups in global MS. A, osteoarthritis dataset; B, chronic low back pain dataset; C, fibromyalgia dataset. CLBP, chronic low back pain; HC, healthy controls; OA, osteoarthritis.

Regional MS had an approximately normal distribution over all 68 cortical regions (after regressing age, sex, and intracranial volume) in both patients and healthy controls from all 3 data sets (Fig. 2—upper panel). We did not find any significant case–control differences in global MS in any of the 3 data sets (OA: T(75) = 0.98, *P*_unc_ = 0.33; CLBP: T(61) = −0.56, *P*_unc_ = 0.58, FM: T(39) = −0.84, *P*_unc_ = 0.41) (Fig. 2—lower panel).

### 3.3. Differences in regional morphometric similarity in patients with chronic pain syndromes as compared with healthy controls

**Figure 3.**
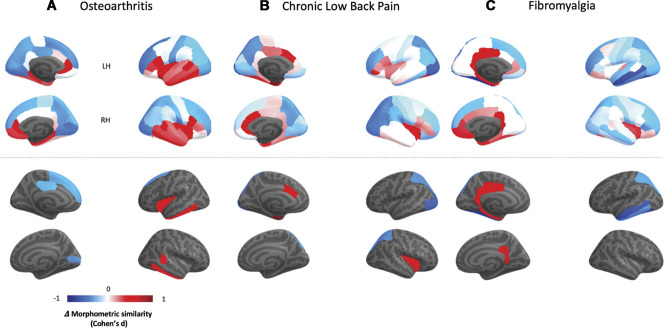
Case–control differences in regional morphometric similarity. In this figure, we present cortical maps of the distribution of Cohen d effect size quantifying case–control differences in regional morphometric similarity (Δ morphometric similarity) for each condition. In the upper panel, we present unthresholded maps. In the lower panel, we present only regions where we found case–control differences for a threshold of *P* < 0.05, uncorrected. Note that none of these regions survived FDR correction for the total number of regions tested within each condition. A, osteoarthritis dataset; B, chronic low back pain dataset; C, fibromyalgia dataset. FDR, false discovery rate.

The cortical maps in Figure 3—upper panel summarize the distribution of case–control changes in cortical MS for each chronic pain condition. In Figure 3—lower panel, we highlight only regions with case–control differences significant at *P* < 0.05, uncorrected (none of these regions survived FDR correction). In the OA data set, we found decreases in MS in the left superior frontal gyrus, right pericalcarine cortex and in the left posterior cingulate, and increases in the left insula and inferior temporal gyrus, and in the right bank of the superior temporal sulcus and right inferior temporal gyrus. In the CLBP data set, we found decreases in MS in the left and right superior parietal gyri and left lateral occipital cortex; and increases in the left entorhinal cortex and caudal anterior cingulate, and in the right insula. In the FM data set, we found decreases in the left superior parietal, medial, and inferior temporal and fusiform gyrus and increases in the left and right isthmus of the cingulate, left posterior cingulate, and entorhinal and parahippocampal cortices. Changes in regional MS correlated positively between conditions (Supplementary Figure S3, available at http://links.lww.com/PAIN/B508), suggesting the existence of a shared pattern of regional MS changes across the 3 conditions. To further support the existence of this pattern, we performed a principal component analysis on the regional MS changes of the 3 conditions, finding that the first PC explained most variance (64.45%) in case–control changes across the 3 conditions (PC1).

Because we used 5 different cortical features to estimate MS (gray matter volume, surface area, cortical thickness, Gaussian curvature and mean curvature), we tested for differential contributions of single cortical features to the observed regional MS changes by recomputing the condition-specific MS change maps excluding each individual cortical feature at a time before MS calculation. We then determined which of these single-feature exclusions most changed the topography of the observed MS changes by identifying the leave-one-feature-out map that correlated the least with the total map. This leave-one-out procedure showed that cortical thickness was the feature that most contributed to the topography of observed regional MS changes across the 3 conditions (Supplementary Figures S4 and S5, available at http://links.lww.com/PAIN/B508). Yet, all leave-one-out maps were positively correlated in all the 3 conditions (Supplementary Figure S4, available at http://links.lww.com/PAIN/B508).

Chronic pain is often comorbid with MDD.^8^ Several preclinical and clinical studies have found considerable overlaps between chronic pain–induced and depression-induced neuroplasticity.^80^ To investigate whether the regional MS changes we report here might predominantly reflect neuroplasticity associated with mood alterations other than chronic pain per se, we used another openly available data set including high resolution structural brain data from unmedicated patients with MDD and healthy controls.^50^ We used these data to define the pattern of changes in regional MS associated with MDD and to investigate whether changes in regional MS in the MDD data set can predict those we observed during chronic pain. We found a considerably different pattern of MS changes in unmedicated MDD patients, as compared with never depressed healthy controls (Supplementary Figure S6A, available at http://links.lww.com/PAIN/B508). Morphometric similarity changes in the MDD data set did not correlate with MS changes in OA and correlated negatively with MS changes in CLBP and FM. We also found a negative correlation between MS changes in MDD and PC1 capturing the cross-condition pattern of changes (Supplementary Figure S6B, available at http://links.lww.com/PAIN/B508). Hence, the pattern of MS changes we report here for patients with chronic pain is unlikely to simply reflect neuroplastic changes associated with comorbid MDD.

### 3.4. Mapping case–control differences in regional morphometric similarity to established patterns of cytoarchitectonic cortical organization

**Figure 4.**
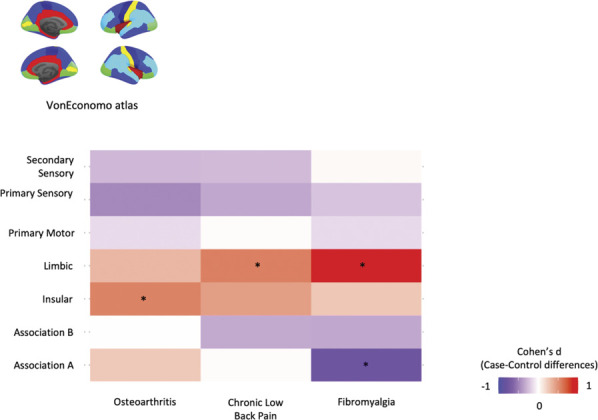
Mapping case–control differences in regional morphometric similarity to established patterns of cytoarchitectonic cortical organization. To help us to contextualize the case–control differences in regional morphometric similarity we observed for the different chronic pain conditions, we mapped them in relation to the von Economo Atlas of the cortex classified by the cytoarchitectonic criteria. For each subject, we quantified MS within each parcel of these atlases (after regressing out age, sex, and intracranial volume) and then performed case–control comparisons using independent sample t tests. The colours in the tile plots represent the Cohen d effect size of case–control differences for each condition. *Highlights significant case–control differences, for *P* < 0.05, uncorrected. MS, morphometric similarity.

To help us contextualize the case–control differences in regional MS we observed for the different chronic pain conditions, we mapped them onto well-established patterns of cytoarchitectonic organization of the cortex as defined by the *von Economo* Atlas of the cortex.^95^ In OA, we found an increase in MS in the insular cortex (*P* < 0.05, uncorrected). In both CLBP and FM, we found increases in MS in the limbic cortex (*P* < 0.05, uncorrected). We also found decreases in association cortex A in FM (*P* < 0.05, uncorrected) (Fig. 4B and Supplementary Table S1, available at http://links.lww.com/PAIN/B508). None of these changes survived FDR correction.

### 3.5. “Hub susceptibility”: associations between case–control differences in regional morphometric similarity and regional morphometric similarity in healthy controls

**Figure 5.**
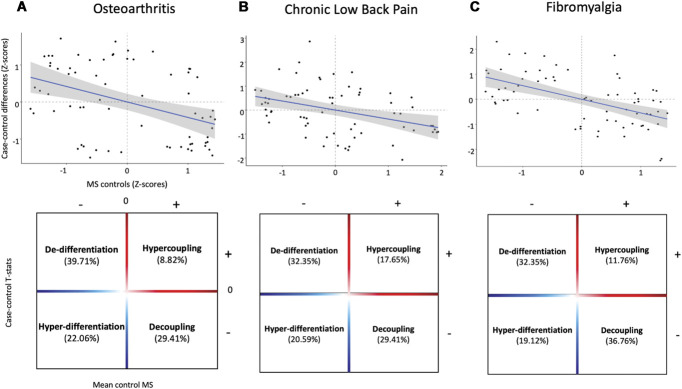
Chronic pain is associated with decoupling of morphometric similarity “hubs” and dedifferentiation of highly differentiated regions. In the upper panel, we present scatterplots depicting significant negative associations between case–control differences in regional morphometric similarity and the mean morphometric similarity of each region in healthy controls. In the lower panel, we subdivided these scatter plots in 4 quadrants according to the relationship between the distribution of case–control differences and mean regional morphometric similarity in healthy controls. The upper left quadrant includes regions of low MS in healthy controls (highly differentiated from the rest of the cortex) that increase their MS with the rest of the cortex during chronic pain (dedifferentiation). The upper right quadrant includes regions of high MS in healthy controls (highly connected with the rest of the cortex) that increase their MS during chronic pain (hypercoupling). The lower left quadrant includes regions of low MS in healthy controls that decrease their MS during chronic pain (hyperdifferentiation). The lower right quadrant includes region of high MS in healthy controls that decrease their MS during chronic pain (ecoupling). Across chronic pain conditions, we observed a predominant pattern of increases in regional MS in regions of low MS (dedifferentiation) and decreases in regions of high MS (decoupling) in healthy controls. Significance was assessed with spatial permutation testing. A, osteoarthritis dataset; B, chronic low back pain dataset; C, fibromyalgia dataset. MS, morphometric similarity.

Previous studies have shown that highly connected “hub” regions are the most likely to show reduced connectivity in disease as measured in functional magnetic resonance imaging and diffusion tensor imaging (DTI) brain networks.^14^ Given the tight positive association between axonal connectivity and MS, these highly connected “hub” regions map to regions with constitutive high MS. Hence, here we also tested this “hub susceptibility” model by investigating relationships between case–control changes in regional MS and the typical pattern of regional MS distribution in healthy controls. Across the 3 data sets, we found significant negative correlations between these 2 variables (OA—Spearman Rho = −0.397, *P*_spin_ = 0.001; CLBP—Spearman Rho = −0.379, *P*_spin_ = 0.003; FM—Spearman Rho = −0.522, *P*_spin_ = 1.7 × 10^−04^) (Fig. 5—upper panel). Moreover, we found that most regional increases map to regions typically showing low MS in controls, whereas most regional decreases map to regions of high MS in controls (Fig. 5—lower panel). Altogether, these findings support the idea that chronic pain is associated with decoupling of MS “hubs” and dedifferentiation of highly differentiated regions.

### 3.6. Transcriptomic correlates of cortical morphometric similarity remodelling during chronic pain

**Figure 6.**
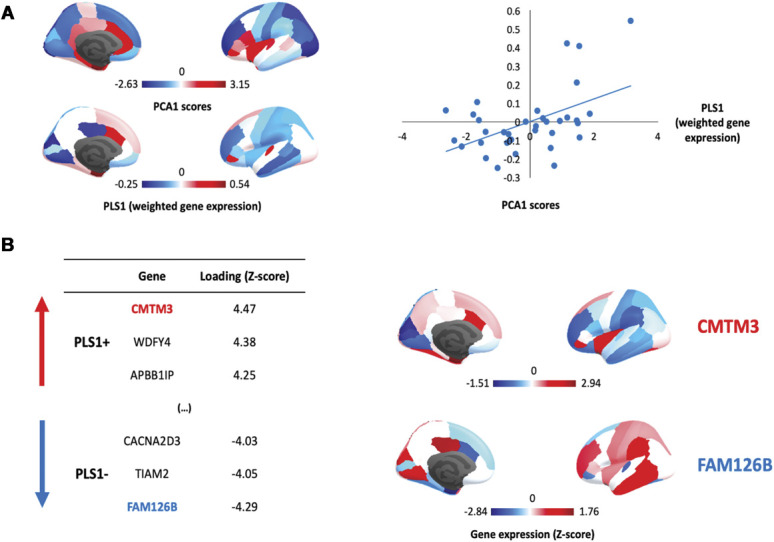
Gene expression profiles related to cross-condition changes in regional morphometric similarity during chronic pain. (A) We summarized case–control changes in regional morphometric similarity (MS) across chronic pain conditions using principal component analysis (PCA). The first component (PC1) explained the large majority of variance 64.45% and was the only component with an eigenvalue > 1. The cortical distribution of the scores of PC1 is depicted in the upper part of panel A. In the lower part, we show the cortical distribution of PLS1 scores summarizing the regional weighted expression of genes associated with cross-condition changes in regional MS during chronic pain. In the scatter plot on the right, we depict a significant positive correlation between PLS1 gene expression weights and the PC1 scores summarizing case–control regional MS differences across conditions. In panel B, we present the top 3 genes positively and negatively associated with PCA1, ranked by the respective loading into PLS1. Loading (Z-score) refers to the weight of each gene in PLS1. Genes with positive weights are highly expressed in regions where MS increases in patients, whereas genes with negative weights are highly expressed in regions where MS decreases. In the right part of panel B, we provide cortical maps summarizing the regional distribution of the top genes with the highest (CMTM3) or lowest (FAM126B) weights in PLS1.

We investigated associations between cross-condition changes in MS during chronic pain (PC1) and brain gene expression using PLS. The first PLS component (PLS1) explained the highest proportion of MS changes (24.4%) and did so above chance (*P*_boot_ = 0.003). PLS1 gene expression weights were positively correlated with cross-condition changes in regional MS (*r* = 0.494, *P*_spin_ = 0.013) (Fig. 6, panel A). This positive correlation means that genes positively weighted on PLS1 are highly expressed in regions where MS was increased, whereas negatively weighted genes are highly expressed in regions where MS was decreased in patients.

We found 338 genes with PLS1 weights Z > 3 (which we denoted PLS1+) and 236 genes with Z < −3 (PLS1−) (all *P*_FDR_ < 0.05). The gene with the highest positive weight was the “Chemokine-Like Factor Superfamily Member 3” (CMTM3), a microglia gene related to immune cytokine activity. The gene with the lowest negative weight was the “Family with Sequence Similarity 126, Member B” (FAM126B), which is a part of a complex required to localize phosphatidylinositol 4-kinase to the plasma membrane (Fig. 6B).

### 3.7. Protein–protein networks and gene set enrichment

**Figure 7.**
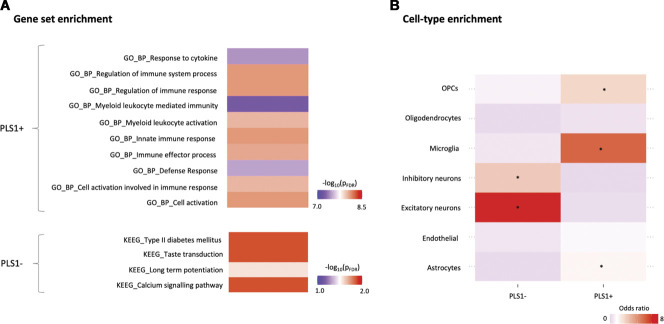
Gene set and cell-type enrichment analyses of the top genes associated with cross-condition changes in regional morphometric similarity during chronic pain. In panel A, we present the results of gene set enrichment analyses on the top genes positively (PLS1+) and negatively (PLS1−) associated with cross-condition changes in regional morphometric similarity during chronic pain, as implemented in the *Functional Mapping and Annotation of Genome-Wide Association Studies* (FUMA) platform. For PLS1+, we present the top 10 Gene Ontology (GO)—biological pathways terms for which we found significant enrichment, ranked by *P*-value after FDR correction. For PLS1−, none of the GO terms survived correction, but we found significant enrichment for 4 terms from the Kyoto Encyclopaedia of Genes and Genomes (KEGG) pathways. Colour scale indicates –log(*P*_FDR_). The full results of the FUMA analysis can be found in supplementary data (available at http://links.lww.com/PAIN/B509, http://links.lww.com/PAIN/B510, http://links.lww.com/PAIN/B511). In panel B, we present the results of a cell-type enrichment analysis, where we investigated whether PLS1+ and PLS1− include overrepresentation of genes typically expressed in specific brain cell types. Enrichment was quantified using odds ratio (OR) and significance calculated with a Fisher exact test. Colour scale indicates OR. Higher ORs (in red) indicate higher enrichment in genes of a certain cell class. The asterisk denotes cell classes for which we found significant enrichment, after correcting for the number of cell types tested (*P_FDR_ < 0.05). FDR, false discovery rate; OPCs, oligodendrocyte precursor cells.

We mapped the network of known interactions between proteins coded by the PLS1+ and PLS1− gene sets (Supplementary Figure S7, available at http://links.lww.com/PAIN/B508). For PLS1+, the resulting network had 303 nodes and 615 edges, more than the 239 edges expected by chance (PPI enrichment *P*-value < 1 × 10^−16^). Using Gene Set Enrichment Analysis, we found enrichment for a number of GO terms—biological pathways broadly mapping to the neuroimmune response axis (Fig. 7A). For PLS1−, the resulting network had 225 nodes and 195 edges, more than the 152 edges expected by chance (PPI enrichment *P*-value 0.0005). Using Gene Set Enrichment Analysis, we did not find enrichment for any GO terms but found 4 terms from the *Kyoto Encyclopedia of Genes and Genomes* (KEGG) pathways that reached significance. Those were “Calcium signalling,” “Long-term potentiation (LTP),” “Taste transduction” and “Type II diabetes mellitus” (Fig. 7A).

### 3.8. Enrichment for transcriptional signatures of canonical brain cell types

We also performed cell-type enrichment analysis using omnibus lists of gene expression in different brain cells of the postmortem human brain, as characterized across 5 different studies. For PLS1+, we found significant enrichment in genes typically expressed in microglia, astrocytes, and oligodendrocytes precursor cells (Fig. 7B and Supplementary Table S4, available at http://links.lww.com/PAIN/B508). For PLS1−, we found significant enrichment in genes typically expressed in excitatory and inhibitory neurons (Fig. 7B and Supplementary Table S2, available at http://links.lww.com/PAIN/B508).

### 3.9. Enrichment for genes related to pain and other brain disorders

Finally, we investigated whether PLS1+ and PLS1− are enriched for pain-related and other brain disorder-related genes as identified in previous studies. For PLS1+, we found enrichment for pain-related genes(OR = 1.40, *P* = 0.04), but not genes related to any of the other brain disorders we tested (Supplementary Table S3, available at http://links.lww.com/PAIN/B508). For PLS1−, we did not find enrichment for pain-related genes (OR = 1.07, *P* = 0.42) but found enrichment for genes associated with epilepsy (OR = 1.54, *P* = 0.02, *P*_FDR_ = 0.09) and MDD (OR = 1.57, *P* = 0.02, *P*_FDR_ = 0.09) (Supplementary Table S3, available at http://links.lww.com/PAIN/B508).

## 4. Discussion

In this article, we uncovered a new pattern of cortical MS remodelling across 3 chronic pain syndromes, which was different from that observed in patients with MDD and points towards the existence of shared disease mechanisms driving cortical remodelling that cut across the boundaries of specific pain syndromes. Furthermore, we demonstrate that cortical MS remodelling in chronic pain spatially correlates with the brain-wide expression of pain-related genes and genes involved in glial immune response and neuronal plasticity, which links putative underlying molecular perturbations with regional vulnerability to brain structural changes. These findings bridge levels to connect genes, cell classes, and biological pathways to in vivo imaging correlates of chronic pain and provide food for beleieved in how future treatment development might be pursued.

Morphometric similarity mapping disclosed a pattern of cortical MS changes across chronic pain syndromes, which generally involved small-to-medium-sized increases in the insula and limbic cortices, and decreases in the occipital, sensorimotor, and frontal cortices. Although most of these changes did not survive correction for multiple comparisons and should be interpreted cautiously, these findings are interesting for several reasons. First, of the various brain regions that have been implicated in the perception of pain, the insula and limbic system are among the ones most consistently reported across studies.^35^ Second, functional and structural alterations in these regions have often been reported in neuroimaging studies of patients with different chronic pain syndromes,^40,58,75,89^ including increases in the connectivity of the insula with nodes of the default-mode network.^9,55,65^ Third, previous studies have further demonstrated that the limbic system plays a key role in the transition from acute-to-chronic pain.^56,57^ Altogether, these aspects reinforce the neuroanatomical plausibility of the pattern in regional MS changes we report in this article.

Morphometric similarity quantifies the correspondence or kinship of 2 cortical areas for multiple macrostructural features that are measurable by MRI.^77^ Hence, high MS between a pair of cortical regions indicates that there is a high degree of correspondence between them for cytoarchitectonic features. This assumption has received empirical support in prior work showing that morphometrically similar cortical regions share patterns of gene coexpression and are more likely to be axonally connected to each other.^77^ Therefore, here, we interpret reduced MS as indicating that there is reduced cytoarchitectonic similarity, or greater cytoarchitectonic differentiation, between these areas and the rest of the cortex, which is probably indicative of reduced anatomical connectivity to and from the less similar, more differentiated cortical areas. On the other hand, increased MS implies increased cytoarchitectonic similarity and, perhaps, axonal connectivity with the rest of the cortex. In line with previousfunctional magnetic resonance imaging and DTI brain networks studies have demonstrated that “hub” regions are more likely to be disturbed and reduce their connectivity in the presence of brain disease,^14^ we found negative associations between regional MS in healthy controls and MS changes in patients with chronic pain. Therefore, it is not implausible that the decreases in MS during chronic pain we describe here might reflect an overall pattern of decreases in axonal connectivity of “hub” regions with the rest of the cortex, as observed in other brain disorders. The reverse, ie, increased connectivity, might drive increases in MS during chronic pain. Nevertheless, we cannot exclude that either increases or decreases in MS might simply reflect local changes in cytoarchitectonics or even a combination of local tissue changes and connectivity.

In an attempt of connecting these MS changes during chronic pain to the gene expression and cellular pathways potentially explaining regional vulnerability to those changes, we used PLS to identify the weighted combination of genes in the whole transcriptome that has a cortical expression map most similar to the cortical map of cross-condition case–control MS differences we derived for patients with chronic pain. Further reinforcing the relationship of this subset of genes with pain, we found enrichment in PLS1+ for pain-related genes but not genes related to other brain disorders. In PLS1−, we did not find enrichment for pain-related genes but found enrichment for genes related to epilepsy and MDD. This last finding is in keep with the idea that chronic pain might share neurobiological pathways with epilepsy^18,68,83^ and depression^80^ and matches well with the clinical observation that antiepileptic drugs^81^ and antidepressants^27^ also improve pain in patients with chronic pain syndromes. We also characterized further the top genes positively and negatively associated MS changes in chronic pain by conducting agnostic gene set enrichment analyses and enrichment for genes associated with different classes of brain cell types. In PLS1+, we found predominance of genes related to the glial immune response and highly expressed in microglia, astrocytes, and oligodendrocyte precursor cells; although in PLS1−, we found predominance of genes related to calcium signalling and LTP, which are highly expressed in excitatory and inhibitory neurons. Altogether, these findings suggest that the constitutive distribution of genes involved in glial immune response and neuronal plasticity, both key elements of the current pathophysiological models of chronic pain,^37,101^ can explain variance in the regional vulnerability to MS cortical remodelling during chronic pain.

How could engagement of these biological and cellular pathways lead to the patterned MS changes we report here? Our imaging transcriptomics findings for PLS1+ suggest that neuroinflammation, with neuronal loss, synapse removal, and glial proliferation,^1^ might drive loss of cortex differentiation during chronic pain.^36,44^ This hypothesis matches well with recent preclinical models highlighting the role of neuroinflammation for neuronal sensitization of pain pathways, at both spinal and brain levels.^29,37,38^ In humans, the neuroinflammation hypothesis has received direct support from positron emission tomography studies showing increased binding of ligands for the 18-kDa translocator protein, currently used as a marker of neuroinflammation and glial activation in the brain,^2–4,54,87^ in the spinal cord, and nerve roots^2^ of patients with chronic pain as compared with healthy controls. On the other hand, our transcriptomic—imaging association findings for PLS1− identify synaptic plasticity of excitatory and inhibitory neuronal populations as a potential driver of the increase in the MS of regions with constitutive low differentiation we report here.^44^ Maladaptive neuronal plasticity, with dysfunctional regulation of the cortical E/I balance has been suggested to contribute to chronic pain.^44,60,70,72^ Shifting of the E/I towards hyperexcitability,^73,88^ as a consequence of either enhanced excitation or reduced inhibition,^49,53^ is believed to augment central pain processing.^44,60^ One key idea around this model is that peripheral injury triggers plastic changes or LTP in the cortical synapses.^13^ Long-term potentiation promotes the formation of synapses and remodelling of dendritic spine substructures,^98^ which compartmentalize calcium.^42,98^ Hence, it is possible that complexification of the synaptic structure, reflecting long-lasting plastic changes in synaptic plasticity, might manifest as decreased MS. However, we should also acknowledge that neuroinflammatory and synaptic processes in the central nervous system tend to complement each other.^63,100^ Therefore, assuming that increases and decreases in MS during chronic pain might involve mutually exclusive biological processes is likely simplistic. Future studies combining ex vivo MRI and histological examinations of the postmortem human brain of patients with chronic pain would be helpful in testing this transcriptomic regional vulnerability model further.

Our study has some limitations worth noting. First, our case–control data sets are relatively small, which might have affected our statistical power to detect small differences (particularly in the context of stringent correction for the number of regions examined). Therefore, future studies with larger sample sizes would be important to assess the replicability of our findings. Second, in the absence of multimodal data, we calculated MS using only 5 parameters from the T1-weighted images of each participant. This might have reduced the precision in estimating MS. Future studies attempting to replicate our findings should ideally use multiple imaging modalities (ie, DTI). Third, although still a general limitation of the field and not of this specific work, the whole-brain gene expression data derive only from 6 *postmortem* adult brains (mean age = 43 years) and include data in the right hemisphere from 2 donors, which led us to exclude MS changes in the right hemisphere for the transcriptomic association analyses. By using constitutive gene expression in a small cohort of 6 postmortem brains to infer associations with neuroimaging markers acquired in different cohorts, we are assuming that regional gene expression is a conserved canonical signature that generalizes well beyond the brain samples included in the AHBA. Although we focused our analyses on probes that were selected to maximize differential stability across donors, 6 postmortem brains are insufficient to make strong claims about the stability of gene expression across brains in humans. Third, we pooled data from 3 different cohorts of patients with different chronic pain syndromes that were collected using different protocols and setups. This aspect poses limitations for investigating condition-specific changes in MS, which are likely to exist and might be interesting to pursue. Moreover, even within the boundaries of a specific chronic pain syndrome, it is likely that different pathophysiological mechanisms are in play in different patients.^64,82,97^ This within-group heterogeneity is an aspect we did not deal with in this study but that future studies should take into consideration. Fourth, the data sets have varied, limited clinical information available, making it difficult to assess the clinical significance of the MS phenotype. Moreover, patients in the different studies were assessed using different clinical tools and detailed characterizations of each cohort were not available (ie, whether different patients might have received different treatments before enrolment). This lack of detailed and comparable data raises the question of whether our patients' cohorts were matched for important clinical variables, such as pain duration or disability. Differences in these factors, if existent, might have contributed to accentuate differences in MS between different chronic pain conditions. Ideally, future studies should aim to recruit, assess, and test all patients under the same protocol to minimize methodological heterogeneity. Fifth, whether MS changes are permanent or might be reversible after treatment is a question that we did not examine but that should be investigated in future longitudinal studies, given that previous studies have reported structural changes in the brain of patients with chronic pain that reverted after treatment.^30,74,79^ Finally, although our findings are suggestive of a potential contribution of neuroimmune responses and neural plasticity to changes in MS, our correlational approach does not allow us to infer causality. This could be investigated further in longitudinal studies examining whether pharmacologic modulation of either pathway might attenuate the changes in MS we report here.

In summary, our study describes a new pattern of cortical MS remodelling across 3 chronic pain syndromes and identifies factors related to the glial immune response and imbalances in neuronal plasticity as candidates for molecular and cellular mechanisms conferring vulnerability to divergent tails of these cortical changes. Altogether, our data indicate that cortical MS remodelling in chronic pain entails a shared component of disease mechanisms that goes beyond specific clinical syndromes boundaries and might involve disruption of multiple elements of the cellular architecture of the brain which is unlikely to be efficiently targeted by current one-size-fits-all treatments. Ultimately, these findings highlight that developing new effective therapeutic approaches to the brain pathology that accompanies chronic pain might require a multitarget approach modulating both glial function and neuronal plasticity.

## Conflict of interest statement

The authors declare no competing interests. This article represents independent research.

## Appendix A. Supplemental digital content

Supplemental digital content associated with this article can be found online at http://links.lww.com/PAIN/B511, http://links.lww.com/PAIN/B508, http://links.lww.com/PAIN/B509 and http://links.lww.com/PAIN/B510.

## Supplementary Material

## References

[1] Aktas O Ullrich O Infante-Duarte C Nitsch R Zipp F. Neuronal damage in brain inflammation. Arch Neurol 2007;64:185–9. 17296833 10.1001/archneur.64.2.185

[2] Albrecht DS Ahmed SU Kettner NW Borra RJH Cohen-Adad J Deng H Houle TT Opalacz A Roth SA Melo MFV Chen L Mao J Hooker JM Loggia ML Zhang Y. Neuroinflammation of the spinal cord and nerve roots in chronic radicular pain patients. PAIN 2018;159:968–77. 29419657 10.1097/j.pain.0000000000001171 PMC5908728

[3] Albrecht DS Forsberg A Sandstrom A Bergan C Kadetoff D Protsenko E Lampa J Lee YC Hoglund CO Catana C Cervenka S Akeju O Lekander M Cohen G Halldin C Taylor N Kim M Hooker JM Edwards RR Napadow V Kosek E Loggia ML. Brain glial activation in fibromyalgia - a multi-site positron emission tomography investigation. Brain Behav Immun 2019;75:72–83. 30223011 10.1016/j.bbi.2018.09.018 PMC6541932

[4] Albrecht DS Mainero C Ichijo E Ward N Granziera C Zurcher NR Akeju O Bonnier G Price J Hooker JM Napadow V Loggia ML Hadjikhani N. Imaging of neuroinflammation in migraine with aura: a [(11)C]PBR28 PET/MRI study. Neurology 2019;92:e2038–50. 30918090 10.1212/WNL.0000000000007371 PMC6511078

[5] Alexander-Bloch A Giedd JN Bullmore E. Imaging structural co-variance between human brain regions. Nat Rev Neurosci 2013;14:322–36. 23531697 10.1038/nrn3465 PMC4043276

[6] Alexander-Bloch A Raznahan A Bullmore E Giedd J. The convergence of maturational change and structural covariance in human cortical networks. J Neurosci 2013;33:2889–99. 23407947 10.1523/JNEUROSCI.3554-12.2013 PMC3711653

[7] Arnatkeviciute A Fulcher BD Fornito A. A practical guide to linking brain-wide gene expression and neuroimaging data. Neuroimage 2019;189:353–67. 30648605 10.1016/j.neuroimage.2019.01.011

[8] Bair MJ Robinson RL Katon W Kroenke K. Depression and pain comorbidity: a literature review. Arch Intern Med 2003;163:2433–45. 14609780 10.1001/archinte.163.20.2433

[9] Baliki MN Mansour AR Baria AT Apkarian AV. Functional reorganization of the default mode network across chronic pain conditions. PLoS One 2014;9:e106133. 25180885 10.1371/journal.pone.0106133 PMC4152156

[10] Baliki MN Schnitzer TJ Bauer WR Apkarian AV. Brain morphological signatures for chronic pain. PLoS One 2011;6:e26010. 22022493 10.1371/journal.pone.0026010 PMC3192794

[11] Carr DB. “Pain is a public health problem”—what does that mean and why should we care? Pain Med 2016;17:626–7. 27052886 10.1093/pm/pnw045

[12] Chen Q Heinricher MM. Descending control mechanisms and chronic pain. Curr Rheumatol Rep 2019;21:13. 30830471 10.1007/s11926-019-0813-1

[13] Chen T Koga K Descalzi G Qiu S Wang J Zhang LS Zhang ZJ He XB Qin X Xu FQ Hu J Wei F Huganir RL Li YQ Zhuo M. Postsynaptic potentiation of corticospinal projecting neurons in the anterior cingulate cortex after nerve injury. Mol pain 2014;10:33. 24890933 10.1186/1744-8069-10-33 PMC4060852

[14] Crossley NA Mechelli A Scott J Carletti F Fox PT McGuire P Bullmore ET. The hubs of the human connectome are generally implicated in the anatomy of brain disorders. Brain 2014;137:2382–95. 25057133 10.1093/brain/awu132 PMC4107735

[15] Dale AM Fischl B Sereno MI. Cortical surface-based analysis. I. Segmentation and surface reconstruction. Neuroimage 1999;9:179–94. 9931268 10.1006/nimg.1998.0395

[16] Darmanis S Sloan SA Zhang Y Enge M Caneda C Shuer LM Hayden Gephart MG Barres BA Quake SR. A survey of human brain transcriptome diversity at the single cell level. Proc Natl Acad Sci U S A 2015;112:7285–90. 26060301 10.1073/pnas.1507125112 PMC4466750

[17] Das S McCaffrey PG Talkington MW Andrews NA Corlosquet S Ivinson AJ Clark T. Pain research forum: application of scientific social media frameworks in neuroscience. Front Neuroinform 2014;8:21. 24653693 10.3389/fninf.2014.00021 PMC3949323

[18] De Caro C Di Cesare Mannelli L Branca JJV Micheli L Citraro R Russo E De Sarro G Ghelardini C Calignano A Russo R. Pain modulation in WAG/Rij epileptic Rats (A genetic model of absence epilepsy): effects of biological and pharmacological histone deacetylase inhibitors. Front Pharmacol 2020;11:549191. 33343343 10.3389/fphar.2020.549191 PMC7745735

[19] De Pauw R Aerts H Siugzdaite R Meeus M Coppieters I Caeyenberghs K Cagnie B. Hub disruption in patients with chronic neck pain: a graph analytical approach. PAIN 2020;161:729–41. 31764388 10.1097/j.pain.0000000000001762

[20] Desikan RS Segonne F Fischl B Quinn BT Dickerson BC Blacker D Buckner RL Dale AM Maguire RP Hyman BT Albert MS Killiany RJ. An automated labeling system for subdividing the human cerebral cortex on MRI scans into gyral based regions of interest. Neuroimage 2006;31:968–80. 16530430 10.1016/j.neuroimage.2006.01.021

[21] DeSouza DD Woldeamanuel YW Sanjanwala BM Bissell DA Bishop JH Peretz A Cowan RP. Altered structural brain network topology in chronic migraine. Brain Struct Funct 2020;225:161–72. 31792696 10.1007/s00429-019-01994-7

[22] Donnelly CR Andriessen AS Chen G Wang K Jiang C Maixner W Ji RR. Central nervous system targets: glial cell mechanisms in chronic pain. Neurotherapeutics 2020;17:846–60. 32820378 10.1007/s13311-020-00905-7 PMC7609632

[23] Dorner TE. Pain and chronic pain epidemiology : implications for clinical and public health fields. Wiener klinische Wochenschrift 2018;130:1–3. 10.1007/s00508-017-1301-0 29270720

[24] Dworkin RH Turk DC Wyrwich KW Beaton D Cleeland CS Farrar JT Haythornthwaite JA Jensen MP Kerns RD Ader DN Brandenburg N Burke LB Cella D Chandler J Cowan P Dimitrova R Dionne R Hertz S Jadad AR Katz NP Kehlet H Kramer LD Manning DC McCormick C McDermott MP McQuay HJ Patel S Porter L Quessy S Rappaport BA Rauschkolb C Revicki DA Rothman M Schmader KE Stacey BR Stauffer JW von Stein T White RE Witter J Zavisic S. Interpreting the clinical importance of treatment outcomes in chronic pain clinical trials: IMMPACT recommendations. J Pain 2008;9:105–21. 18055266 10.1016/j.jpain.2007.09.005

[25] Farmer MA Baliki MN Apkarian AV. A dynamic network perspective of chronic pain. Neurosci Lett 2012;520:197–203. 22579823 10.1016/j.neulet.2012.05.001 PMC3377811

[26] Fauchon C Meunier D Rogachov A Hemington KS Cheng JC Bosma RL Osborne NR Kim JA Hung PS Inman RD Davis KD. Sex differences in brain modular organization in chronic pain. PAIN 2021;162:1188–1200. 33044396 10.1097/j.pain.0000000000002104

[27] Ferreira GE McLachlan AJ Lin CC Zadro JR Abdel-Shaheed C O'Keeffe M Maher CG. Efficacy and safety of antidepressants for the treatment of back pain and osteoarthritis: systematic review and meta-analysis. BMJ 2021;372:m4825. 33472813 10.1136/bmj.m4825 PMC8489297

[28] Goulas A Uylings HB Hilgetag CC. Principles of ipsilateral and contralateral cortico-cortical connectivity in the mouse. Brain Struct Funct 2017;222:1281–95. 27497948 10.1007/s00429-016-1277-y

[29] Guan Z Kuhn JA Wang X Colquitt B Solorzano C Vaman S Guan AK Evans-Reinsch Z Braz J Devor M Abboud-Werner SL Lanier LL Lomvardas S Basbaum AI. Injured sensory neuron-derived CSF1 induces microglial proliferation and DAP12-dependent pain. Nat Neurosci 2016;19:94–101. 26642091 10.1038/nn.4189 PMC4703328

[30] Gwilym SE Filippini N Douaud G Carr AJ Tracey I. Thalamic atrophy associated with painful osteoarthritis of the hip is reversible after arthroplasty: a longitudinal voxel-based morphometric study. Arthritis Rheum 2010;62:2930–40. 20518076 10.1002/art.27585

[31] Habib N Avraham-Davidi I Basu A Burks T Shekhar K Hofree M Choudhury SR Aguet F Gelfand E Ardlie K Weitz DA Rozenblatt-Rosen O Zhang F Regev A. Massively parallel single-nucleus RNA-seq with DroNc-seq. Nat Methods 2017;14:955–8. 28846088 10.1038/nmeth.4407 PMC5623139

[32] Hawrylycz M Miller JA Menon V Feng D Dolbeare T Guillozet-Bongaarts AL Jegga AG Aronow BJ Lee CK Bernard A Glasser MF Dierker DL Menche J Szafer A Collman F Grange P Berman KA Mihalas S Yao Z Stewart L Barabasi AL Schulkin J Phillips J Ng L Dang C Haynor DR Jones A Van Essen DC Koch C Lein E. Canonical genetic signatures of the adult human brain. Nat Neurosci 2015;18:1832–44. 26571460 10.1038/nn.4171 PMC4700510

[33] Hawrylycz MJ Lein ES Guillozet-Bongaarts AL Shen EH Ng L Miller JA van de Lagemaat LN Smith KA Ebbert A Riley ZL Abajian C Beckmann CF Bernard A Bertagnolli D Boe AF Cartagena PM Chakravarty MM Chapin M Chong J Dalley RA David Daly B Dang C Datta S Dee N Dolbeare TA Faber V Feng D Fowler DR Goldy J Gregor BW Haradon Z Haynor DR Hohmann JG Horvath S Howard RE Jeromin A Jochim JM Kinnunen M Lau C Lazarz ET Lee C Lemon TA Li L Li Y Morris JA Overly CC Parker PD Parry SE Reding M Royall JJ Schulkin J Sequeira PA Slaughterbeck CR Smith SC Sodt AJ Sunkin SM Swanson BE Vawter MP Williams D Wohnoutka P Zielke HR Geschwind DH Hof PR Smith SM Koch C Grant SGN Jones AR. An anatomically comprehensive atlas of the adult human brain transcriptome. Nature 2012;489:391–9. 22996553 10.1038/nature11405 PMC4243026

[34] Hocking LJ Generation S Morris AD Dominiczak AF Porteous DJ Smith BH. Heritability of chronic pain in 2195 extended families. Eur J Pain 2012;16:1053–63. 22337623 10.1002/j.1532-2149.2011.00095.x

[35] Iannetti GD Mouraux A. From the neuromatrix to the pain matrix (and back). Exp Brain Res 2010;205:1–12. 20607220 10.1007/s00221-010-2340-1

[36] Jafari M Schumacher AM Snaidero N Ullrich Gavilanes EM Neziraj T Kocsis-Jutka V Engels D Jurgens T Wagner I Weidinger JDF Schmidt SS Beltran E Hagan N Woodworth L Ofengeim D Gans J Wolf F Kreutzfeldt M Portugues R Merkler D Misgeld T Kerschensteiner M. Phagocyte-mediated synapse removal in cortical neuroinflammation is promoted by local calcium accumulation. Nat Neurosci 2021;24:355–67. 33495636 10.1038/s41593-020-00780-7

[37] Ji RR Nackley A Huh Y Terrando N Maixner W. Neuroinflammation and central sensitization in chronic and widespread pain. Anesthesiology 2018;129:343–66. 29462012 10.1097/ALN.0000000000002130 PMC6051899

[38] Ji RR Xu ZZ Gao YJ. Emerging targets in neuroinflammation-driven chronic pain. Nat Rev Drug Discov 2014;13:533–48. 24948120 10.1038/nrd4334 PMC4228377

[39] Jiang BC Liu T Gao YJ. Chemokines in chronic pain: cellular and molecular mechanisms and therapeutic potential. Pharmacol Ther 2020;212:107581. 32450191 10.1016/j.pharmthera.2020.107581

[40] Kang DH Son JH Kim YC. Neuroimaging studies of chronic pain. Korean J Pain 2010;23:159–65. 20830260 10.3344/kjp.2010.23.3.159 PMC2935976

[41] Kaplan CM Schrepf A Vatansever D Larkin TE Mawla I Ichesco E Kochlefl L Harte SE Clauw DJ Mashour GA Harris RE. Functional and neurochemical disruptions of brain hub topology in chronic pain. PAIN 2019;160:973–83. 30763287 10.1097/j.pain.0000000000001480 PMC6424595

[42] Kim HY Lee KY Lu Y Wang J Cui L Kim SJ Chung JM Chung K. Mitochondrial Ca(2+) uptake is essential for synaptic plasticity in pain. J Neurosci 2011;31:12982–91. 21900577 10.1523/JNEUROSCI.3093-11.2011 PMC3179262

[43] King DJ Wood AG. Clinically feasible brain morphometric similarity network construction approaches with restricted magnetic resonance imaging acquisitions. Netw Neurosci 2020;4:274–91. 32181419 10.1162/netn_a_00123 PMC7069065

[44] Kuner R Flor H. Structural plasticity and reorganisation in chronic pain. Nat Rev Neurosci 2017;18:113. 28704354 10.1038/nrn.2017.5

[45] Labus JS Dinov ID Jiang Z Ashe-McNalley C Zamanyan A Shi Y Hong JY Gupta A Tillisch K Ebrat B Hobel S Gutman BA Joshi S Thompson PM Toga AW Mayer EA. Irritable bowel syndrome in female patients is associated with alterations in structural brain networks. PAIN 2014;155:137–49. 24076048 10.1016/j.pain.2013.09.020 PMC4100785

[46] Lacroix-Fralish ML Ledoux JB Mogil JS. The Pain Genes Database: an interactive web browser of pain-related transgenic knockout studies. PAIN 2007;131:3.e1–4. 17574758 10.1016/j.pain.2007.04.041

[47] Lake BB Chen S Sos BC Fan J Kaeser GE Yung YC Duong TE Gao D Chun J Kharchenko PV Zhang K. Integrative single-cell analysis of transcriptional and epigenetic states in the human adult brain. Nat Biotechnol 2018;36:70–80. 29227469 10.1038/nbt.4038 PMC5951394

[48] Larkin TE Kaplan CM Schrepf A Ichesco E Mawla I Harte SE Mashour GA Clauw DJ Harris RE. Altered network architecture of functional brain communities in chronic nociplastic pain. Neuroimage 2021;226:117504. 33293261 10.1016/j.neuroimage.2020.117504

[49] Lefaucheur JP Drouot X Menard-Lefaucheur I Keravel Y Nguyen JP. Motor cortex rTMS restores defective intracortical inhibition in chronic neuropathic pain. Neurology 2006;67:1568–74. 17101886 10.1212/01.wnl.0000242731.10074.3c

[50] Lepping RJ Atchley RA Chrysikou E Martin LE Clair AA Ingram RE Simmons WK Savage CR. Neural processing of emotional musical and nonmusical stimuli in depression. PLoS One 2016;11:e0156859. 27284693 10.1371/journal.pone.0156859 PMC4902194

[51] Li M Santpere G Imamura Kawasawa Y Evgrafov OV Gulden FO Pochareddy S Sunkin SM Li Z Shin Y Zhu Y Sousa AMM Werling DM Kitchen RR Kang HJ Pletikos M Choi J Muchnik S Xu X Wang D Lorente-Galdos B Liu S Giusti-Rodriguez P Won H de Leeuw CA Pardinas AF BrainSpan C Psych EC Psych EDS Hu M Jin F Li Y Owen MJ O'Donovan MC Walters JTR Posthuma D Reimers MA Levitt P Weinberger DR Hyde TM Kleinman JE Geschwind DH Hawrylycz MJ State MW Sanders SJ Sullivan PF Gerstein MB Lein ES Knowles JA Sestan N. Integrative functional genomic analysis of human brain development and neuropsychiatric risks. Science 2018;362:eaat7615. 30545854 10.1126/science.aat7615 PMC6413317

[52] Li J Seidlitz J Suckling J Fan F Ji GJ Meng Y Yang S Wang K Qiu J Chen H Liao W. Cortical structural differences in major depressive disorder correlate with cell type-specific transcriptional signatures. Nat Commun 2021;12:1647. 33712584 10.1038/s41467-021-21943-5 PMC7955076

[53] Lim M Roosink M Kim JS Kim DJ Kim HW Lee EB Kim HA Chung CK. Disinhibition of the primary somatosensory cortex in patients with fibromyalgia. PAIN 2015;156:666–74. 25630027 10.1097/j.pain.0000000000000096

[54] Loggia ML Chonde DB Akeju O Arabasz G Catana C Edwards RR Hill E Hsu S Izquierdo-Garcia D Ji RR Riley M Wasan AD Zurcher NR Albrecht DS Vangel MG Rosen BR Napadow V Hooker JM. Evidence for brain glial activation in chronic pain patients. Brain 2015;138:604–15. 25582579 10.1093/brain/awu377 PMC4339770

[55] Loggia ML Kim J Gollub RL Vangel MG Kirsch I Kong J Wasan AD Napadow V. Default mode network connectivity encodes clinical pain: an arterial spin labeling study. PAIN 2013;154:24–33. 23111164 10.1016/j.pain.2012.07.029 PMC3534957

[56] Makary MM Polosecki P Cecchi GA DeAraujo IE Barron DS Constable TR Whang PG Thomas DA Mowafi H Small DM Geha P. Loss of nucleus accumbens low-frequency fluctuations is a signature of chronic pain. Proc Natl Acad Sci U S A 2020;117:10015–23. 32312809 10.1073/pnas.1918682117 PMC7211984

[57] Mansour AR Baliki MN Huang L Torbey S Herrmann KM Schnitzer TJ Apkarian VA. Brain white matter structural properties predict transition to chronic pain. PAIN 2013;154:2160–8. 24040975 10.1016/j.pain.2013.06.044 PMC3799881

[58] Martucci KT Ng P Mackey S. Neuroimaging chronic pain: what have we learned and where are we going? Future Neurol 2014;9:615–26. 28163658 10.2217/FNL.14.57 PMC5289824

[59] Meloto CB Benavides R Lichtenwalter RN Wen X Tugarinov N Zorina-Lichtenwalter K Chabot-Dore AJ Piltonen MH Cattaneo S Verma V Klares R III Khoury S Parisien M Diatchenko L. Human pain genetics database: a resource dedicated to human pain genetics research. PAIN 2018;159:749–63. 29300278 10.1097/j.pain.0000000000001135

[60] Mhalla A de Andrade DC Baudic S Perrot S Bouhassira D. Alteration of cortical excitability in patients with fibromyalgia. PAIN 2010;149:495–500. 20356675 10.1016/j.pain.2010.03.009

[61] Mills SEE Nicolson KP Smith BH. Chronic pain: a review of its epidemiology and associated factors in population-based studies. Br J Anaesth 2019;123:e273–83. 31079836 10.1016/j.bja.2019.03.023 PMC6676152

[62] Morgan SE Seidlitz J Whitaker KJ Romero-Garcia R Clifton NE Scarpazza C van Amelsvoort T Marcelis M van Os J Donohoe G Mothersill D Corvin A Pocklington A Raznahan A McGuire P Vertes PE Bullmore ET. Cortical patterning of abnormal morphometric similarity in psychosis is associated with brain expression of schizophrenia-related genes. Proc Natl Acad Sci U S A 2019;116:9604–9. 31004051 10.1073/pnas.1820754116 PMC6511038

[63] Mottahedin A Ardalan M Chumak T Riebe I Ek J Mallard C. Effect of neuroinflammation on synaptic organization and function in the developing brain: implications for neurodevelopmental and neurodegenerative disorders. Front Cell Neurosci 2017;11:190. 28744200 10.3389/fncel.2017.00190 PMC5504097

[64] Murphy SL Lyden AK Phillips K Clauw DJ Williams DA. Subgroups of older adults with osteoarthritis based upon differing comorbid symptom presentations and potential underlying pain mechanisms. Arthritis Res Ther 2011;13:R135. 21864381 10.1186/ar3449 PMC3239378

[65] Napadow V LaCount L Park K As-Sanie S Clauw DJ Harris RE. Intrinsic brain connectivity in fibromyalgia is associated with chronic pain intensity. Arthritis Rheum 2010;62:2545–55. 20506181 10.1002/art.27497 PMC2921024

[66] Niederberger E. Novel insights into molecular mechanisms of chronic pain. Cells 2020;9:2220. 10.3390/cells9102220 PMC7601569 33019536

[67] Nielsen CS Knudsen GP Steingrimsdottir OA. Twin studies of pain. Clin Genet 2012;82:331–40. 22823509 10.1111/j.1399-0004.2012.01938.x

[68] Panczyk K Golda S Waszkielewicz A Zelaszczyk D Gunia-Krzyzak A Marona H. Serotonergic system and its role in epilepsy and neuropathic pain treatment: a review based on receptor ligands. Curr Pharm Des 2015;21:1723–40. 25412650 10.2174/1381612821666141121114917

[69] Pando-Naude V Barrios FA Alcauter S Pasaye EH Vase L Brattico E Vuust P Garza-Villarreal EA. Functional connectivity of music-induced analgesia in fibromyalgia. Sci Rep 2019;9:15486. 31664132 10.1038/s41598-019-51990-4 PMC6820536

[70] Petrou M Pop-Busui R Foerster BR Edden RA Callaghan BC Harte SE Harris RE Clauw DJ Feldman EL. Altered excitation-inhibition balance in the brain of patients with diabetic neuropathy. Acad Radiol 2012;19:607–12. 22463961 10.1016/j.acra.2012.02.004 PMC3374728

[71] Pinero J Bravo A Queralt-Rosinach N Gutierrez-Sacristan A Deu-Pons J Centeno E Garcia-Garcia J Sanz F Furlong LI. DisGeNET: a comprehensive platform integrating information on human disease-associated genes and variants. Nucleic Acids Res 2017;45:D833–9. 27924018 10.1093/nar/gkw943 PMC5210640

[72] Pomares FB Roy S Funck T Feier NA Thiel A Fitzcharles MA Schweinhardt P. Upregulation of cortical GABAA receptor concentration in fibromyalgia. PAIN 2020;161:74–82. 31569142 10.1097/j.pain.0000000000001707 PMC6940028

[73] Qu XX Cai J Li MJ Chi YN Liao FF Liu FY Wan Y Han JS Xing GG. Role of the spinal cord NR2B-containing NMDA receptors in the development of neuropathic pain. Exp Neurol 2009;215:298–307. 19046970 10.1016/j.expneurol.2008.10.018

[74] Rodriguez-Raecke R Niemeier A Ihle K Ruether W May A. Brain gray matter decrease in chronic pain is the consequence and not the cause of pain. J Neurosci 2009;29:13746–50. 19889986 10.1523/JNEUROSCI.3687-09.2009 PMC6666725

[75] Schmidt-Wilcke T. Neuroimaging of chronic pain. Best Pract Res Clin Rheumatol 2015;29:29–41. 26266997 10.1016/j.berh.2015.04.030

[76] Seidlitz J Nadig A Liu S Bethlehem RAI Vertes PE Morgan SE Vasa F Romero-Garcia R Lalonde FM Clasen LS Blumenthal JD Paquola C Bernhardt B Wagstyl K Polioudakis D de la Torre-Ubieta L Geschwind DH Han JC Lee NR Murphy DG Bullmore ET Raznahan A. Transcriptomic and cellular decoding of regional brain vulnerability to neurogenetic disorders. Nat Commun 2020;11:3358. 32620757 10.1038/s41467-020-17051-5 PMC7335069

[77] Seidlitz J Vasa F Shinn M Romero-Garcia R Whitaker KJ Vertes PE Wagstyl K Kirkpatrick Reardon P Clasen L Liu S Messinger A Leopold DA Fonagy P Dolan RJ Jones PB Goodyer IM Consortium N Raznahan A Bullmore ET. Morphometric similarity networks detect microscale cortical organization and predict inter-individual cognitive variation. Neuron 2018;97:231–47.e237. 29276055 10.1016/j.neuron.2017.11.039 PMC5763517

[78] Seifert F Maihofner C. Central mechanisms of experimental and chronic neuropathic pain: findings from functional imaging studies. Cell Mol Life Sci 2009;66:375–90. 18791842 10.1007/s00018-008-8428-0 PMC11131450

[79] Seminowicz DA Wideman TH Naso L Hatami-Khoroushahi Z Fallatah S Ware MA Jarzem P Bushnell MC Shir Y Ouellet JA Stone LS. Effective treatment of chronic low back pain in humans reverses abnormal brain anatomy and function. J Neurosci 2011;31:7540–50. 21593339 10.1523/JNEUROSCI.5280-10.2011 PMC6622603

[80] Sheng J Liu S Wang Y Cui R Zhang X. The link between depression and chronic pain: neural mechanisms in the brain. Neural Plast 2017;2017:9724371. 28706741 10.1155/2017/9724371 PMC5494581

[81] Sidhu HS Sadhotra A. Current status of the new antiepileptic drugs in chronic pain. Front Pharmacol 2016;7:276. 27610084 10.3389/fphar.2016.00276 PMC4996999

[82] Spahr N Hodkinson D Jolly K Williams S Howard M Thacker M. Distinguishing between nociceptive and neuropathic components in chronic low back pain using behavioural evaluation and sensory examination. Musculoskelet Sci Pract 2017;27:40–8. 28637600 10.1016/j.msksp.2016.12.006 PMC5329124

[83] Stevenson SB. Epilepsy and migraine headache: is there a connection? J Pediatr Health Care 2006;20:167–71. 16675377 10.1016/j.pedhc.2005.10.014

[84] Szklarczyk D Morris JH Cook H Kuhn M Wyder S Simonovic M Santos A Doncheva NT Roth A Bork P Jensen LJ von Mering C. The STRING database in 2017: quality-controlled protein-protein association networks, made broadly accessible. Nucleic Acids Res 2017;45:D362–8. 27924014 10.1093/nar/gkw937 PMC5210637

[85] Taneja A Della Pasqua O Danhof M. Challenges in translational drug research in neuropathic and inflammatory pain: the prerequisites for a new paradigm. Eur J Clin Pharmacol 2017;73:1219–36. 28894907 10.1007/s00228-017-2301-8 PMC5599481

[86] Tetreault P Mansour A Vachon-Presseau E Schnitzer TJ Apkarian AV Baliki MN. Brain connectivity predicts placebo response across chronic pain clinical trials. PLoS Biol 2016;14:e1002570. 27788130 10.1371/journal.pbio.1002570 PMC5082893

[87] Torrado-Carvajal A Toschi N Albrecht DS Chang K Akeju O Kim M Edwards RR Zhang Y Hooker JM Duggento A Kalpathy-Cramer J Napadow V Loggia ML. Thalamic neuroinflammation as a reproducible and discriminating signature for chronic low back pain. PAIN 2021;162:1241–9. 33065737 10.1097/j.pain.0000000000002108 PMC7969370

[88] Toyoda H Zhao MG Ulzhofer B Wu LJ Xu H Seeburg PH Sprengel R Kuner R Zhuo M. Roles of the AMPA receptor subunit GluA1 but not GluA2 in synaptic potentiation and activation of ERK in the anterior cingulate cortex. Mol Pain 2009;5:46. 19664265 10.1186/1744-8069-5-46 PMC2734546

[89] Tracey I Bushnell MC. How neuroimaging studies have challenged us to rethink: is chronic pain a disease? J Pain 2009;10:1113–20. 19878862 10.1016/j.jpain.2009.09.001

[90] Tsuda M Shigemoto-Mogami Y Koizumi S Mizokoshi A Kohsaka S Salter MW Inoue K. P2X4 receptors induced in spinal microglia gate tactile allodynia after nerve injury. Nature 2003;424:778–83. 12917686 10.1038/nature01786

[91] Vasa F Seidlitz J Romero-Garcia R Whitaker KJ Rosenthal G Vertes PE Shinn M Alexander-Bloch A Fonagy P Dolan RJ Jones PB Goodyer IM consortium N Sporns O Bullmore ET. Adolescent tuning of association cortex in human structural brain networks. Cereb Cortex 2018;28:281–94. 29088339 10.1093/cercor/bhx249 PMC5903415

[92] Wada A Shizukuishi T Kikuta J Yamada H Watanabe Y Imamura Y Shinozaki T Dezawa K Haradome H Abe O. Altered structural connectivity of pain-related brain network in burning mouth syndrome-investigation by graph analysis of probabilistic tractography. Neuroradiology 2017;59:525–32. 28361345 10.1007/s00234-017-1830-2

[93] Watanabe K Taskesen E van Bochoven A Posthuma D. Functional mapping and annotation of genetic associations with FUMA. Nat Commun 2017;8:1826. 29184056 10.1038/s41467-017-01261-5 PMC5705698

[94] Watkins LR Martin D Ulrich P Tracey KJ Maier SF. Evidence for the involvement of spinal cord glia in subcutaneous formalin induced hyperalgesia in the rat. PAIN 1997;71:225–35. 9231865 10.1016/s0304-3959(97)03369-1

[95] Whitaker KJ Vertes PE Romero-Garcia R Vasa F Moutoussis M Prabhu G Weiskopf N Callaghan MF Wagstyl K Rittman T Tait R Ooi C Suckling J Inkster B Fonagy P Dolan RJ Jones PB Goodyer IM Consortium N Bullmore ET. Adolescence is associated with genomically patterned consolidation of the hubs of the human brain connectome. Proc Natl Acad Sci U S A 2016;113:9105–10. 27457931 10.1073/pnas.1601745113 PMC4987797

[96] Writing Committee for the Attention-Deficit/Hyperactivity D, Autism Spectrum D, Bipolar D, Major Depressive D, Obsessive-Compulsive D, and Schizophrenia EWG; Patel Y Parker N Shin J Howard D French L Thomopoulos SI Pozzi E Abe Y Abe C Anticevic A Alda M Aleman A Alloza C Alonso-Lana S Ameis SH Anagnostou E McIntosh AA Arango C Arnold PD Asherson P Assogna F Auzias G Ayesa-Arriola R Bakker G Banaj N Banaschewski T Bandeira CE Baranov A Bargallo N Bau CHD Baumeister S Baune BT Bellgrove MA Benedetti F Bertolino A Boedhoe PSW Boks M Bollettini I Del Mar Bonnin C Borgers T Borgwardt S Brandeis D Brennan BP Bruggemann JM Bulow R Busatto GF Calderoni S Calhoun VD Calvo R Canales-Rodriguez EJ Cannon DM Carr VJ Cascella N Cercignani M Chaim-Avancini TM Christakou A Coghill D Conzelmann A Crespo-Facorro B Cubillo AI Cullen KR Cupertino RB Daly E Dannlowski U Davey CG Denys D Deruelle C Di Giorgio A Dickie EW Dima D Dohm K Ehrlich S Ely BA Erwin-Grabner T Ethofer T Fair DA Fallgatter AJ Faraone SV Fatjo-Vilas M Fedor JM Fitzgerald KD Ford JM Frodl T Fu CHY Fullerton JM Gabel MC Glahn DC Roberts G Gogberashvili T Goikolea JM Gotlib IH Goya-Maldonado R Grabe HJ Green MJ Grevet EH Groenewold NA Grotegerd D Gruber O Gruner P Guerrero-Pedraza A Gur RE Gur RC Haar S Haarman BCM Haavik J Hahn T Hajek T Harrison BJ Harrison NA Hartman CA Whalley HC Heslenfeld DJ Hibar DP Hilland E Hirano Y Ho TC Hoekstra PJ Hoekstra L Hohmann S Hong LE Hoschl C Hovik MF Howells FM Nenadic I Jalbrzikowski M James AC Janssen J Jaspers-Fayer F Xu J Jonassen R Karkashadze G King JA Kircher T Kirschner M Koch K Kochunov P Kohls G Konrad K Kramer B Krug A Kuntsi J Kwon JS Landen M Landro NI Lazaro L Lebedeva IS Leehr EJ Lera-Miguel S Lesch KP Lochner C Louza MR Luna B Lundervold AJ MacMaster FP Maglanoc LA Malpas CB Portella MJ Marsh R Martyn FM Mataix-Cols D Mathalon DH McCarthy H McDonald C McPhilemy G Meinert S Menchon JM Minuzzi L Mitchell PB Moreno C Morgado P Muratori F Murphy CM Murphy D Mwangi B Nabulsi L Nakagawa A Nakamae T Namazova L Narayanaswamy J Jahanshad N Nguyen DD Nicolau R O'Gorman Tuura RL O'Hearn K Oosterlaan J Opel N Ophoff RA Oranje B Garcia de la Foz VO Overs BJ Paloyelis Y Pantelis C Parellada M Pauli P Pico-Perez M Picon FA Piras F Piras F Plessen KJ Pomarol-Clotet E Preda A Puig O Quide Y Radua J Ramos-Quiroga JA Rasser PE Rauer L Reddy J Redlich R Reif A Reneman L Repple J Retico A Richarte V Richter A Rosa PGP Rubia KK Hashimoto R Sacchet MD Salvador R Santonja J Sarink K Sarro S Satterthwaite TD Sawa A Schall U Schofield PR Schrantee A Seitz J Serpa MH Setien-Suero E Shaw P Shook D Silk TJ Sim K Simon S Simpson HB Singh A Skoch A Skokauskas N Soares JC Soreni N Soriano-Mas C Spalletta G Spaniel F Lawrie SM Stern ER Stewart SE Takayanagi Y Temmingh HS Tolin DF Tomecek D Tordesillas-Gutierrez D Tosetti M Uhlmann A van Amelsvoort T van der Wee NJA van der Werff SJA van Haren NEM van Wingen GA Vance A Vazquez-Bourgon J Vecchio D Venkatasubramanian G Vieta E Vilarroya O Vives-Gilabert Y Voineskos AN Volzke H von Polier GG Walton E Weickert TW Weickert CS Weideman AS Wittfeld K Wolf DH Wu MJ Yang TT Yang K Yoncheva Y Yun JY Cheng Y Zanetti MV Ziegler GC Franke B Hoogman M Buitelaar JK van Rooij D Andreassen OA Ching CRK Veltman DJ Schmaal L Stein DJ van den Heuvel OA Turner JA van Erp TGM Pausova Z Thompson PM Paus T. Virtual histology of cortical thickness and shared neurobiology in 6 psychiatric disorders. JAMA Psychiatry 2021;78:47–63. 32857118 10.1001/jamapsychiatry.2020.2694 PMC7450410

[97] Wylde V Hewlett S Learmonth ID Dieppe P. Persistent pain after joint replacement: prevalence, sensory qualities, and postoperative determinants. PAIN 2011;152:566–72. 21239114 10.1016/j.pain.2010.11.023

[98] Yuste R Bonhoeffer T. Morphological changes in dendritic spines associated with long-term synaptic plasticity. Annu Rev Neurosci 2001;24:1071–89. 11520928 10.1146/annurev.neuro.24.1.1071

[99] Zhang Y Sloan SA Clarke LE Caneda C Plaza CA Blumenthal PD Vogel H Steinberg GK Edwards MS Li G Duncan JA III Cheshier SH Shuer LM Chang EF Grant GA Gephart MG Barres BA. Purification and characterization of progenitor and mature human astrocytes reveals transcriptional and functional differences with mouse. Neuron 2016;89:37–53. 26687838 10.1016/j.neuron.2015.11.013 PMC4707064

[100] Zhou LJ Peng J Xu YN Zeng WJ Zhang J Wei X Mai CL Lin ZJ Liu Y Murugan M Eyo UB Umpierre AD Xin WJ Chen T Li M Wang H Richardson JR Tan Z Liu XG Wu LJ. Microglia are indispensable for synaptic plasticity in the spinal dorsal horn and chronic pain. Cell Rep 2019;27:3844–59. e3846. 31242418 10.1016/j.celrep.2019.05.087 PMC7060767

[101] Zhuo M. Cortical excitation and chronic pain. Trends Neurosci 2008;31:199–207. 18329111 10.1016/j.tins.2008.01.003

